# A multilevel analysis of trends and predictors associated with teenage pregnancy in Zambia (2001–2018)

**DOI:** 10.1186/s12978-023-01567-2

**Published:** 2023-01-18

**Authors:** Million Phiri, Mwewa E. Kasonde, Nkuye Moyo, Milika Sikaluzwe, Simona Simona

**Affiliations:** 1 Department of Population Studies, School of Humanities and Social Sciences, University of Zambia, Lusaka, Zambia; 2 Demography and Population Studies Programme, Schools of Public Health and Social Sciences, University of the Witwatersrand, Johannesburg, South Africa; 3 Department of Social Work and Sociology, School of Humanities and Social Sciences, University of Zambia, Lusaka, Zambia

**Keywords:** Teenage girls, Teenage pregnancy, Trends, Multilevel analysis, Zambia

## Abstract

**Background:**

Teenage pregnancy remains a major social and public health challenge in developing countries especially sub-Saharan Africa (SSA) where prevalence rates are still increasing. Even if considerable effort has been made over the years to study determining factors of teenage pregnancy in SSA, few studies have looked at the trends and associated factors over a longer period. Furthermore, no known study has focussed on both individual and contextual factors influencing teenage pregnancy in Zambia. This study, thus sought to fill this gap in knowledge by simultaneously investigating trends of teenage pregnancy as well as its individual and contextual determining factors.

**Methods:**

A total pooled weighted sample of 10,010 teenagers (in the age group 15–19) from four waves of the Zambia Demographic and Health Surveys were extracted. Using bivariate analysis, we investigated the trends of teenage pregnancy between 2001 and 2018. Separate multilevel logistic regression models were fitted on pooled teenage pregnancy data in relation to several individual and contextual level factors. Both fixed and random effects were produced. Bayesian parameter estimates were produced using *lme4* package in R statistical programming environment.

**Results:**

Results of the trends of teenage pregnancy in Zambia have shown an overall decrease of 2% between 2001 and 2018. Almost all the socioeconomic and demographic variables were consistently associated with teenage pregnancy (p < 0.001) in a bivariate analysis across the four survey. In multilevel analysis, the odds of being pregnant were higher for teenagers who were employed (aOR = 1.21, 95% CI: 1.02–1.42), married (aOR = 7.71, 95% CI: 6.31–9.52) and those with knowledge of ovulation period (aOR = 1.58, 95% CI: 1.34–1.90). On the other hand, belonging to households in high wealth quintiles, being literate, exposure to mass-media family planning messages and delayed sexual debut were associated with decreased odds of teenage pregnancy.

**Conclusion:**

The study shows that teenage pregnancy remains a social and public health challenge in Zambia as the country has seen little decrease in the prevalence over the years under consideration. Factors associated with teenage pregnancy include marital status, and employment, knowledge of ovulation period, wealth quintile, sexual debut and exposure to mass-media family planning messaging. Concerted effort must be made to improve literacy levels, reduce poverty and enhance sexual health promotion through the mass media in view of cultural norms, which may prevent parents and children from discussion sexual education topics thus exacerbate the vice.

**Supplementary Information:**

The online version contains supplementary material available at 10.1186/s12978-023-01567-2.

## Plain language summary

Teenage pregnancy continues to be a major social and public health challenge in developing countries, particularly in Sub-Saharan Africa (SSA), where prevalence rates are on the rise. Even if considerable effort has been made over the years to study determining factors of teenage pregnancy in SSA, few studies have looked at the trends and associated factors over a longer period. This study in Zambia, thus sought to fill this gap in knowledge by simultaneously investigating trends of teenage pregnancy as well as its individual and contextual determining factors. Here, we used a pooled dataset from Zambia’s 2001 to 2018 DHS to run separate multilevel logistic regression models to examine several individual and contextual level factors influencing teenage pregnancy. Findings show that trends of teenage pregnancy in Zambia have shown an overall decrease of only 2% between 2001 and 2018. Almost all the socioeconomic and demographic variables were consistently associated with teenage pregnancy. The study shows that teenage pregnancy remains a social and public health challenge in Zambia as the country has seen little decrease in the prevalence over the years under consideration. Concerted effort must be made to improve literacy levels, reduce poverty and enhance sexual health promotion through the mass media in view of cultural norms, which may prevent parents and children from discussion sexual education topics thus exacerbate the vice.

**Supplementary Information:**

The online version contains supplementary material available at 10.1186/s12978-023-01567-2.

## Introduction

Teenage pregnancy is defined as the occurrence of pregnancy among young girls between 10 and 19 years [1, 2]. It is a major social and public health problem for both developing and developed countries. The prevalence rate of teenage pregnancy among adolescents aged 15–19 in Africa is 18.8% while that of sub-Saharan Africa (SSA) is around 19.3% [3]. The drivers of teenage pregnancy are several and multifaceted and these include poverty, inadequate education and employment opportunities, gender inequalities, rural residence, poor access and low use of contraceptives and inadequate sexual and reproductive health (SRH) information among others [3–6]. Marital status is another major factor associated with teenage pregnancies. In many societies, girls are under immense pressure to get married, and the social status that is associated with childbirth means that married young girls have subsequent pressure to bear children [7]. It is for this reason that about 90% of all teenage pregnancies occur within marriage [8]. Additionally, low contraceptive use has been observed as a key contributing factor to high teenage pregnancies in SSA. Although contraceptive use among teenagers increased from 7.6% in 1996 to 10.9% in 2014 in Zambia [9], the prevalence remains low. There are rural–urban differentials in the use of contraception among teenagers in Zambia, with teenagers in rural areas having higher contraceptive prevalence rates compared to their counterparts living in urban areas [9–11].

Teenage pregnancy comes with a great deal of health risks and complications for both the teenagers themselves and their babies. The World Health Organization (WHO) estimates that pregnancy and childbirth complications are the leading cause of death among girls aged 15–19 years globally, with low- and middle-income countries accounting for 99% of global maternal deaths of women aged 15–49 years [12, 13]. Further, some 3.9 million unsafe abortions among girls aged 15–19 occur each year, contributing to maternal mortality, morbidity and lasting health problems [14]. This phenomena may be exacerbated during the era of the COVID-19 pandemic that disrupted provision of SRH services among teenagers and young mothers [15]. Additionally, babies born from teenage mothers face higher risk of low birth weight, preterm delivery and severe neonatal conditions. Younger mothers are also prone to violence by an intimate partner [13] which can expose them to poor maternal health outcomes. At a global level, population growth tends to be more rapid when women are exposed to early childbirth, as this will lengthen the reproductive period and increase the fertility rate [17].

In Zambia, just like most parts of SSA, the prevalence of teenage pregnancy is high and a prominent issue in social, political, and cultural discourse. In 2018, teenage pregnancy was estimated at 29.2% [18], making Zambia one of the countries with significantly high prevalence of teenage pregnancy among SSA countries. The prevalence, however, has notably been in decline from 31.6% in 1992 to 29.2% in 2018 [18] albeit with significant within-country variations. The prevalence is significantly higher in rural areas (37%) compared to urban areas (19%) [18]. Teenage pregnancy in Zambia is reported to be twice as high as that of the SSA region [2, 19].

The government of Zambia, with support from cooperating partners such as UNFPA, UNESCO, UNICEF and the World Bank, has been implementing various programmes and strategies over the past ten years aimed at reducing the prevalence of teenage pregnancy [20, 21]. In 2014, the government, in coordination with the Ministry of Education, introduced an ambitious nation-wide programme for comprehensive sexuality education (CSE) to be implemented into ordinary school activities by teachers. The CSE curriculum is firmly based in a discourse of enhancing access to sexual and reproductive information and rights among school going adolescents [22–24]. With support from the World Bank in 2015, the Keeping Girls in School programme was launched to reduce secondary school drop-out rates among adolescents' girls in marginalised communities [25]. Furthermore, the launch of the Adolescent Health Strategy (2017–2021) was aimed at addressing health policy and programme bottlenecks that impede adolescents' access to sexual reproductive health services [26].

Although the prevalence of teenage pregnancy appears to be falling overtime, it remains unacceptably high. These levels of teenage pregnancy signify the fact that more needs to be done to stimulate a further reduction in levels of teenage pregnancy among this demographic at a health policy level. Doing so requires a holistic understanding of factors associated with teenage pregnancy. This understanding of factors affecting adolescent pregnancy is much more lacking when we consider individual and contextual factors. Therefore, this study was designed to fill in knowledge gaps as well as make policy suggestions aimed at reducing teenage pregnancy rates in the country. This study aimed to investigate factors associated with teenage pregnancy in Zambia. The study also sought to establish if there were community variations in teenage pregnancy. Unlike a similar study conducted in Zambia [4], this study incorporates trends and contextual analyses, as well as uses a complex and comprehensive dataset. These objectives were accomplished by making use of the nationally representative sample from the Zambia Demographic and Health Survey (ZDHS) data gathered between 2001 and 2018. To establish if the factors associated with teenage pregnancy vary between communities, we conducted a two-level multilevel analysis in view of the hierarchical ZDHS data structure of individual women who are nested within clusters.

## Methods and data

### Study data source

This study is a secondary micro data analysis of the existing national level data from the Zambia Demographic and Health Survey (ZDHS) program. The ZDHS is a nationally representative household survey conducted by the Zambia Statistics Agency with support from global partners including the ICF International and United States Agency for International Development (USAID). The DHS uses two-stage sampling to select enumeration areas (EAs) in the first stage and households in the second stage. The nature of DHS data allows for comparisons between variables over time, thus allowing monitoring changes in the indicators of variables of interest in different geographical areas [27]. Women ages 15–49 years of selected households who had accepted to participate in the study were also enrolled to participate in the survey. A detailed description of the methods used in the DHS are published elsewhere [18]. For this study, we extracted all relevant variables from the women data files (individual recode) in the 2001–02, 2007, 2013–14 and 2018 ZDHS data sets. The data analysed in this study relate to teenage girls ages 15–19 years. The four DHSs used in the analysis captured a pooled weighted sample of 10,010 teenagers. The distribution of sample sizes per survey year is reported in Table 2.

### Study variables

#### Outcome variable

The outcome variable of interest for this study was teenage pregnancy. Teenage pregnancy prevalence is defined as “Percentage of teenage girls (ages 15–19 years) who have had a live birth or are currently pregnant with their first child” [28, 29]. In this study, teenage pregnancy was created as a composite variable comprising of: teenage girls who ever gave birth and those who were pregnant with their first child at the time of the survey [28]. The dependent variable was coded as binary with code “1” representing teenage girls who have ever given birth or were pregnant for the first time and coded as “0” if otherwise. Data in this study only includes current or prior pregnancies.

#### Independent variables

Based on existing literature, we identified potential factors that could influence teenage pregnancy in Zambia at two levels (individual and community). The individual level variables included socio-economic, demographic, health and environmental factors. DHS reference materials and data collection forms were used to identify the individual level independent variables presented in (Table 1). The variable exposure to mass-media family planning (FP) messages is a composite variable that was created from three DHS variables (received FP messages on radio, television (TV), newspapers). Multiple correspondence analysis was used to analyse nominal categorical data to create a composite variable that would measure the proportion of teenagers who had access to at least one mass media source. This variable was constructed as a binary variable (yes/no).

**Table 1:** Definition of individual level predictor variables

No	Variable category	Variable name	Definition and measurement
1	Demographic	Age	Age of teenage respondents at time of survey: range from 15 -19 years
2		Marital status	Current marital status of teenager; 1 = Never married; 2 = Married/Living with partner; 3 = Formerly married
3		Age at first sex	Age at which teenagers reported having first sexual encounter; 1 = 10–14 years; 2 = 15–17 years; 3 = 18–19 years
4	Social	Literacy	Ability to read: 1 = Able to read; 2 = Cannot read at all
5		Exposure to mass-media FP messages	Access to mass-media FP messages: 1 = No; 2 = Yes
6		Education level	Education level attained by teenager; 1 = No education; 2 = Primary; 3 = Secondary or Higher
7	Economic	Wealth status	Income classification of households where teenagers belong; 1 = Poorest; 2 = Poor; 3 = Middle; 4 = Rich; 5 = Richest
8		Employment status	Employment status of teenagers: 1 = Employed; 2 = Unemployed
9	Health factors	Knowledge of FP method	Teenagers’ have knowledge of any modern contraceptive method: 0 = Knows no method; 1 = Knows a method
10		Knowledge of ovulation period	Teenagers’ have knowledge of ovulation period: 1 = No; 2 = Yes
11		Visited health facility in last 12-months	Teenagers who visited health facility in past 12 month prior to survey: 1 = No; 2 = Yes

#### Community-level variables

Community-level variables, which were created by aggregating individual-level data into clusters, included community poverty, community education and community knowledge of FP methods, and place of residence. All community-level variables are categorized as ‘low’ or ‘high’ representing the magnitude of the phenomena being studied at the cluster level. Place of residence and geographical region retained original categorizations. Place of residence is one of the criteria utilised in designing the sample to estimate the prevalence of core demographic and health indicators at the national level. It is categorised as ‘rural’ or ‘urban’ and it directly explains community characteristics.

### Statistical analysis

The initial analysis was performed using Stata version 14.2 (Stata Corp. Inc. Texas, USA) taking into consideration survey design, cluster effect and post-stratification weights. Key socio-economic and demographic factors were described and expressed in frequency and percentage distributions over time. Trend analysis of teenage pregnancy was conducted relative to the respective survey years (between 2001 and 2018). Exploratory bivariate analysis was carried out separately for each dataset to assess the association between the prevalence of teenage pregnancy and the selected independent variables outlined in Table 2.

**Table 2:** Description of teenagers by background characteristics, 2001–2018 DHS, Zambia

Background characteristic	2001–02 DHS	2007 DHS	2013–14 DHS	2018 DHS
	N = 1811	N = 1574	N = 3625	N = 3000
Age				
15	365 (20.2%)	364 (23.1%)	740 (20.4%)	653 (21.8%)
16	330 (18.2%)	328 (20.8%)	766 (21.1%)	530 (17.7%)
17	326 (18.0%)	295 (18.7%)	642 (17.7%)	552 (18.4%)
18	417 (23.0%)	293 (18.6%)	745 (20.6%)	722 (24.1%)
19	374 (20.7%)	294 (18.7%)	732 (20.2%)	543 (18.1%)
Residence				
Urban	763 (42.1%)	761 (48.4%)	1740 (48.0%)	1323 (44.1%)
Rural	1048 (57.9%)	813 (51.6%)	1886 (52.0%)	1677 (55.9%)
Educational level				
No education	145 (8.0%)	63 (4.0%)	68 (1.9%)	99 (3.3%)
Primary	1053 (58.1%)	764 (48.5%)	1395 (38.5%)	1283 (42.8%)
Secondary or higher	613 (33.8%)	747 (47.5%)	2158 (59.6%)	1618 (53.9%)
Literacy				
Illiterate	741 (41.1%)	420 (26.7%)	744 (20.7%)	728 (24.3%)
Literate	1063 (58.9%)	1154 (73.3%)	2858 (79.3%)	2272 (75.7%)
Employed				
No	1217 (67.3%)	1271 (80.8%)	2895 (80.4%)	2477 (82.6%)
Yes	593 (32.7%)	303 (19.2%)	705 (19.6%)	523 (17.4%)
Wealth quintile				
Poorest	303 (16.7%)	219 (13.9%)	546 (15.0%)	510 (17.0%)
Poorer	296 (16.3%)	246 (15.6%)	591 (16.3%)	541 (18.0%)
Middle	354 (19.5%)	261 (16.6%)	677 (18.7%)	585 (19.5%)
Richer	397 (21.9%)	385 (24.4%)	802 (22.1%)	655 (21.8%)
Richest	462 (25.5%)	463 (29.4%)	1010 (27.9%)	709 (23.6%)
Marital status				
Never married	1322 (73.0%)	1268 (80.5%)	2950 (81.4%)	2531 (84.4%)
Married	438 (24.2%)	280 (17.8%)	613 (16.9%)	437 (14.6%)
Formerly married	52 (2.9%)	26 (1.7%)	63 (1.7%)	32 (1.1%)
Age at first sex				
Less than 15	281 (31.6%)	167 (26.0%)	392 (25.0%)	359 (24.1%)
15–17	541 (60.9%)	413 (64.4%)	1,015 (64.8%)	971 (65.2%)
18–19	67 (7.5%)	62 (9.6%)	160 (10.2%)	159 (10.7%)

A two-level multilevel binary logistic regression model was applied on pooled data for all the four surveys to assess the effects of several individual and community level factors on teenage pregnancy in Zambia. Adjusted odds ratios (aOR) with corresponding 95% confidence intervals (CI) were reported. Three multilevel logistic models were estimated. Model 1 included only the intercept and the outcome variable. Model 2 included the individual level variables while Model 3 had both the individual and community level variables. All significantly associated factors from the bivariate analyses were included in multilevel analysis (p < 0.05). The multilevel model assesses the probability ${p}_{ij}$ of a teenage woman or teenager $i$ in a community $j$ having a teenage pregnancy. This analysis is represented as:

$$logit {(p}_{ij})= {\beta }_{0}+ {\beta \mathrm{\rm X}}_{ij}+ uj+ \upsilon$$

where ${X}_{ij}$ is a vector of independent variables at individual and community levels. $uj$ is normally distributed with variance ${\sigma }_{u}^{2}$; $\upsilon$ is normally distributed with variance${\sigma }_{u}^{2}$.

In terms of variances used to understand the variations of relationships between communities and the relative effect of community-level variables, the variance partition coefficient (VPC) was used. VPC provides information on the share of variance at each level. The latent method was used to calculate the VPC at each level. It assumes a threshold model, approximating the level 1 variance by ${\pi }^{2}/3(\approx 3.29)$ [30, 31]. The community level variance is calculated as follows:

$${VPC}_{Community}=\frac{{\sigma }_{u}^{2}}{{\sigma }_{u}^{2}+ {\pi }^{2}/3}$$

For model comparisons and assessment of goodness of fit, we used the Bayesian Information Criteria (BIC). Multilevel logistic regression models were implemented using the *lme4* package in the R statistical programming environment [32]. To assess multicollinearity among independent factors, the Variance Inflation Factor (VIF) was used. There were no concerns with multicollinearity in any of the variables (all VIF < 5) (Additional file 1: Table S1).

### Ethics

The study is based on the ZDHS data, which is publicly available on (https://dhsprogram.com/). All data used did not contain any identifying information. The original Zambia DHS 2001–2018 Biomarker and survey protocols were approved by Tropical Disease and Research Center (TDRC) and the Research Ethics Review Board of the Centers for Disease Control and Prevention (CDC) Atlanta.

## Results

### Description of sample characteristics

Table 2 shows the distribution of teenagers included in the analysis by background characteristics across the four survey years. The distribution of teenagers across ages shows that most of them were aged 18 years in the three surveys years (2001–02, 2013–14 and 2018). Rural and urban distribution showed that in all the four survey years, the majority of the teenagers were from rural areas (over 50%). Regarding the highest level of education, majority of respondents had attained primary school level education in the 2001–02 (58.1%) and 2007 (48.5%). While in 2013–14 and 2018 surveys, most teenagers had attained secondary and higher level of education (59.6% and 53.9%), respectively. In terms of employment status, majority of teenagers were out of employment across all the survey years.

Table 2 further shows that in all survey years 2001–02, 2007, 2013–14 and 2018, most of the teenagers had their first sexual debut in the age group 15–17 (60.9%, 64.4% and 64.8, 65.2%, respectively). Majority of the teenagers were never married, ranging from 73% in 2001–02 to 84.4% in 2018.

### Trends in teenage pregnancy in Zambia, 2001–2018

Teenage pregnancy slightly reduced from 32 to 29% in Zambia from 2001 to 2018 (Table 3). Bivariate analysis reveals that many socio-economic and demographic variables (residence, education level, literacy, employment status, wealth status, marital status, knowledge of FP method and visiting health facility in the last 12 months) were consistently associated with teenage pregnancy across the four survey years (p < 0.001)..

**Table 3:** Percentage distribution of teenage pregnancy by background characteristics, 2001 -2018 DHS, Zambia

Background characteristic	2001–2 DHS	2007 DHS	2013–14 DHS	2018 DHS
	N = 1811	N = 1574	N = 3625	N = 3000
	% [95% CI]	% [95% CI]	% [95% CI]	% [95% CI]
Age	***	***	***	***
15	4.5 [2.7,7.3]	5.8 [3.7,9.1]	4.9 [3.4,7.0]	6.4 [4.6,8.8]
16	15.0 [11.1,19.8]	16.2 [12.2,21.1]	11.9 [9.6,14.7]	15.1 [12.1,18.7]
17	33.8 [28.9,39.2]	28.7 [23.1,35.0]	25.7 [21.7,30.1]	30.0 [25.1,35.4]
18	44.2 [28.9,39.2]	41.0 [34.5,47.8]	41.7 [37.4,46.1]	41.9 [37.3,46.6]
19	56.9 [51.6,62.0]	54.6 [48.4,60.7]	58.9 [54.5,63.3]	52.9 [47.6,58.0]
Residence	***	***	***	***
Urban	27.1 [23.1,31.6]	20.4 [16.4,25.1]	20.0 [17.4,23.0]	19.3 [15.7,23.6]
Rural	34.9 [32.1,37.8]	35 [31.2,39.0]	36.4 [33.8,39.0]	37.0 [34.4,39.7]
Education level	***	***	***	***
No education	45.6 [36.8,54.7]	54.3 [42.1,66.1]	53.2 [38.6,67.3]	41.9 [32.0,52.4]
Primary	35.7 [32.7,38.7]	32.9 [29.2,36.7]	35.8 [32.6,39.1]	36.3 [33.4,39.4]
Secondary or higher	21.3 [17.9,25.2]	20.6 [17.1,24.7]	23.0 [20.7,25.4]	22.8 [19.7,26.3]
Literacy	***	***	***	***
Illiterate	41.0 [37.4,44.7]	42.8 [37.5,48.3]	44.1 [39.7,48.5]	47.7 [43.3,52.0]
Literate	25.3 [22.3,28.6]	22.5 [19.5,25.9]	24.4. [22.5,26.5]	23.3 [20.8,26.1]
Employment status	***	***	***	***
Unemployed	24.5 [21.9,27.4]	24.3 [21.3,27.6]	23.8 [21.7,25.9]	24.9 [22.8,27.3]
Employed	46.3 [42.0,50.6]	43.0 [37.2,49.1]	47.5 [43.0,52.0]	49.5 [44.5,54.5]
Wealth quintile	***	***	***	***
Poorest	36.3 [31.4,41.5]	37.2 [30.4,44.5]	44.5 [39.8,49.3]	46.2 [41.5,50.9]
Poorer	40.3 [34.7,46.2]	34.4 [28.2,41.1]	38.5 [34.2,43.1]	38.0 [33.7,42.6]
Middle	34.9 [30.2,39.9]	36.7 [31.3,42.5]	34.5 [30.5,38.7]	35.0 [30.5,39.8]
Richer	36.4 [31.8,41.3]	29.4 [24.5,34.8]	28.2 [24.4,32.3]	27.0 [22.5,32.0]
Richest	16.3 [12.8,20.6]	14.0 [10.0,19.2]	10.3 [7.9,13.4]	7.6 [5.3,10.8]
Marital status	***	***	***	***
Never married	13.1 [11.4,15.1]	14.2 [12.2,16.5]	14.4. [13.0,15.9]	18.1 [16.2,20.2]
Currently married	82.9 [78.9,86.2]	85.0 [79.3,89.3]	91.3 [88.2,93.6]	89.0 [85.3,91.9]
Formerly married	70.4 [56.3,81.5]	81.8 [59.2,93.2]	82.0 [68.7,90.4]	91.2 [65.4,98.3]
Knows ovulation period	ns	**	ns	ns
No	31.0 [28.5,33.7]	26.3 [23.4,29.5]	28.1 [26.0,30.2]	29.1 [26.7,31.6]
Yes	34.6 [28.7,41.2]	36.3 [29.6,43.6]	30.9 [26.5,35.7]	30.0 [24.8,35.9]
Age at first sex	ns	*	ns	***
10–14	51.5 [45.2,57.8]	53.5 [45.3,61.5]	51.9 [46.1,57.6]	65.2 [59.5,70.4]
15–17	52.7 [48.4,57.1]	57.1 [51.9,62.2]	56.2 [52.5,59.8]	59.5 [55.2,63.6]
18–19	36.6 [25.5,49.3]	35.9 [23.8,50.2]	47.2 [38.6,56.0]	41.3 [32.8,50.5]
Knowledge of FP method	***	***	***	***
No	6.2 [3.1,11.9]	11.1 [6.2,19.1]	9.2 [5.2,15.7]	6.7 [3.1,13.8]
Yes	33.4 [30.8,36.1]	29.7 [26.6,32.9]	29.5 [27.5,31.6]	30.5 [28.0,33.0]
Exposure to media FP messages	*	ns	**	*
No	34.0 [31.2,36.9]	29.2 [25.7,32.8]	30.2 [27.9,32.7]	30.4 [28.1,32.8]
Yes	28.4 [24.7,32.3]	25.7 [21.4,30.5]	24.4. [21.4,27.8]	23.5 [18.5,29.2]
Visited health facility last 12 months	***	***	***	***
No	15.8 [13.4,18.6]	18.2 [15.6,21.1]	17.0 [14.9,19.3]	15.2 [12.9,18.0]
Yes	49.4 [45.4,53.4]	55.4 [48.3,62.2]	49.2 [45.6,52.7]	45.6 [42.1,49.2]
Total	31.6 [29.2,34.1]	27.9 [25.1,31.0]	28.5 [26.6,30.6]	29.2 [26.9,31.6]

***p < 0.001; **p < 0.01; *p < 0.05; *ns* non-significant

Teenage pregnancy has been consistently high in rural areas and among those with primary or no education (p < 0.001). Teenage pregnancy has been generally high across the three education level categories (no education, primary and secondary or higher). Among those with no education, prevalence dropped from 54 to 42% in the period 2007 to 2018. Further, teenagers who were illiterate were more likely to become pregnant than those who were literate in all the survey years (p < 0.001). The prevalence increased from 41% in 2001 to 48% in 2018 among the illiterate teens.

Differences in prevalence of teenage pregnancy according to marital status were statistically significant across the four ZDHSs (p < 0.001). Married and formerly married teenagers comprised the highest prevalence of teenage pregnancy compared to those who reported to have never been married. For example, in 2018, 89% of the married teenagers versus 18% of those never married experienced teenage pregnancy. Further, early sexual debut was also found to be associated with a higher risk of teenage pregnancy in 2007 and 2018 ZDHS.

Similarly, study results show variations in prevalence of teenage pregnancy and household wealth status (p < 0.001). Teenagers from poor households had higher rates of teenage pregnancy compared to those from rich households. In 2018, 8% of teens who belonged to richest household compared to 46% from poorest households had experienced teenage pregnancy. The percentage of teens who fell pregnant from poorest households increased over time from 36% in 2001 to 46% in 2018; however, a significant reduction in teenage pregnancy was observed among teens who belonged to richest households (16.2% in 2001 to 7.6% in 2018).

We also found that teenagers who were exposed to media FP messages had lower risks of being pregnant before age 20 in 2001, 2007 and 2018. Trends show a reduction in teenage pregnancy from 28 to 24% between 2001 and 2018 among teenagers who were exposed to media FP messages. However, knowledge of FP method and visiting a health facility in the last 12 months were negatively associated with teenage pregnancy, although trends show a reduction in teenage pregnancy among teenagers who visited the health facility in the last 12 months from 49% in 2001 to 46% in 2018 (Table 3).

### Multilevel analysis of predictors of teenage pregnancy in Zambia 2001–2018

The multilevel analysis results are presented in Table 4 using the odds ratios and 95% confidence intervals (CI) for both the individual and community-level variables. Both the fixed and random effects are reported with appropriate levels of significance. The results are displayed in three models with model 1 being the null model showing results from the random intercept multilevel analysis without individual and community level variables. This is intended to show the level of heterogeneity between clusters and indicate variations in teenage pregnancies attributable to differences in clusters. The VPC of 10.6% [i.e. 0.39/ (0.39 + 3.29)] from the variance component model before all independent variables are controlled for shows significant contribution of community (cluster) level variables to variations in total teenage pregnancies in Zambia.

**Table 4:** Multilevel parameter estimates and adjusted odds of teenage pregnancy, DHS 2001–2018, Zambia

Variables	(N = 10,010)		
	Model 1	Model 2	Model 3
	aOR (95% CI)	aOR (95% CI)	aOR (95% CI)
Intercept	0.41 (0.39,0.43)***	0.06 (0.03,0.12)***	0.06 (0.03, 0.12)***
Survey year			
2001		Ref.	Ref.
2007		2.58 (1.98, 3.34)***	2.57 (1.98, 3.34)***
2014		1.77 (1.42, 2.21)***	1.79 (1.43, 2.23)***
2018		1.55 (1.24, 1.94)***	1.57 (1.26, 1.96)***
Age			
15		Ref.	Ref.
16		1.90 (1.40, 2.58)***	1.89 (1.39, 2.57)***
17		3.33 (2.46, 4.52)***	3.33 (2.45, 4.51)***
18		4.93 (3.65, 6.65)***	4.91 (3.64, 6.73)***
19		8.72 (6.34, 12.00)***	8.72 (6.34, 11.98)***
Educational level			
No education		Ref.	Ref.
Primary		1.09 (0.73, 1.63)^ns^	1.09 (0.73, 1.64)^ns^
Secondary or higher		0.94 (0.59, 1.48)^ns^	0.94 (0.59, 1.51)^ns^
Literacy			
Illiterate		Ref.	Ref.
Literate		0.78 (0.63, 0.97)*	0.78 (0.64, 0.96)*
Employment status			
No		Ref.	Ref.
Yes		1.20 (1.02, 1.41)*	1.21 (1.02, 1.42)*
Wealth quintile			
Poorest		Ref.	Ref.
Poorer		0.85 (0.67, 1.07)	0.84 (0.66, 1.07)
Middle		0.76 (0.60, 0.96)*	0.76 (0.59, 0.96)*
Richer		0.75 (0.59, 0.96)*	0.73 (0.55, 0.96)*
Richest		0.40 (0.30, 0.53)***	0.38 (0.27, 0.52)***
Marital status			
Never married		Ref.	Ref.
Married		7.53 (6.12, 9.26)***	7.71 (6.31, 9.52)***
Formerly married		4.64 (2.79, 7.72)***	4.60 (2.76, 7.66)***
Knows of ovulation period			
No		Ref.	Ref.
Yes		1.60 (1.34, 1.89)***	1.58 (1.34, 1.90)***
Age at first sex			
10–14		Ref.	Ref.
15–17		0.70(0.58,0.83)***	0.69(0.58,83)***
18–19		0.23(0.17,0.31)***	0.22(0.17,0.31)***
Knowledge of FP method			
No		Ref.	Ref.
Yes		1.54 (0.94, 2.56)	1.49 (0.90, 2.45)
Exposure to media FP messages			
No		Ref.	Ref.
Yes		0.69 (0.58, 0.82)***	0.66 (0.55, 0.80)***
Visited health facility last 12 months			
No		Ref.	Ref.
Yes		3.92 (3.41, 4.61)***	3.92 (3.37, 4.57)***
Contextual variables			
Place of residence			
Urban			Ref.
Rural			0.94 (0.76, 1.14)^ns^
Community education			
Low			Ref.
High			0.99 (0.83, 1.18)^ns^
Community poverty			
Low			Ref.
High			1.10 (0.91, 1.33)^ns^
Community media FP exposure			
Low			Ref.
High			0.82 (0.70, 0.96)*
Random effects			
Variance (SE)	0.39 (0.05)	0.33 (0.09)	0.30 (0.13)
VPC (%)	10.60	8.96	8.36
BIC	12,366.0	4972.0	4944.5

***p < 0.001; ** = *p* < 0.01; * = *p* < 0.05; *ns* non-significant, *aOR* adjusted odds ratio, *CI* confidence internal, *SE* standard error, *VPC* variance partition coefficients, *BIC* Bayesian Information Criterion

Table 4 also shows features for model comparisons. After adjusting for the effects of all observable individual and community level variables, about 8.4% of the total unexplained variation in teenage pregnancies in Zambia is attributable to unobserved community level factors. The Bayesian Information Criteria (BIC shows that models, which includes all the individual and community level factors are better fit compared to the single level and the intercept only models. As such, the interpretation of results focused mainly on the model 3, which includes all the independent variables in the study.

The survey year confirms the crude analysis that emerged in the bivariate analysis where teenage pregnancies seemed to increase over time in most of the variables. After adjusting for the influence of all included variables, the odds of teenage pregnancy increased by 2.57 times in 2007 compared to 2001. The increased odds of teenage pregnancy are observed in the subsequent survey years of 2014 and 2018 albeit with relatively reduced odds. Other significant predictors of teenage pregnancy include age, literacy levels, employment status, marital status, knowledge of ovulation period and visiting health facilities. Married teenagers have much higher odds of being pregnant than their unmarried counterparts (aOR = 7.71; 95% CI = 6.31, 9.52, p < 0.001).

Knowledge of ovulation period shows surprising results whereby young women who know the ovulation period are 1.58 times (p < 0.001) more likely to be pregnant than those who do not know. Being literate reduces the odds of teenage pregnancy by 0.78 (p < 0.05) while being employed increases the odds of teenage pregnancy by 1.21 times (p < 0.05). Young women who had initiated sexual activities at a later age of 18–19 had significantly reduced odds (aOR: 0.22; 95% CI = 0.17, 0.31, p < 0.001) of teenage pregnancy compared to those who did so before age 18. Visiting health facilities in the last 12 months does not seem to be a cushion for teenage pregnancy since those who visited health facilities had significantly higher odds of teenage pregnancy than those did not. Exposure to FP messages, however, reduces odds of teenage pregnancy by 0.66. Poverty is also a significant predictor of teenage pregnancy as young women who belong to wealthier households have less odds of having teenage pregnancies compared to those from poorer households. Education level and knowledge of FP methods are not significant predictors of teenage pregnancy in Zambia.

Community level factors were mostly not found to be significantly associated with the odds of teenage pregnancies except for community media FP exposure, exemplifying the importance of mass media in health promotion. The results further show that living in communities with high exposure to FP messages is associated with a reduction in the odds of teenage pregnancy.

## Discussion

This study sought to analyse the trends and factors associated with teenage pregnancy using multilevel modelling of ZDHS data between 2001 and 2018. The study focused on the influence of both individual and community level factors on teenage pregnancy as well as how these factors vary between clusters. Our review of literature reveals that no known comprehensive study of this nature has been conducted in Zambia and thus bolstering the importance of our findings. The significant findings indicate that overall, there is a minimal decrease in teenage pregnancy in Zambia. However, there are apparent disparities in the trends of teenage pregnancies among different groups of teenagers. Teenagers who live in rural areas and are from poorest households had an increase in teenage pregnancy over the four surveys analysed for this study. This finding could be explained by inadequate access to SRH information and low contraception use among young teenager from rural areas and those from poor communities [9, 10, 23, 33]. The increase in the trends of teenage pregnancy was also observed among those illiterate and married/formerly married teenage women. An apparent decline in teenage pregnancy was observed among young women who are exposed to FP messages and for those who know the ovulation period.

At the individual level, we found that marital status was strongly associated with teenage pregnancy. Teenagers that reported being currently married or those living with partners were more likely to be pregnant compared to their never-married counterparts. These findings are consistent with evidence from other previous studies in Zambia [4] and Africa [3]. The relationship between marital status and teenage pregnancy has long been held in literature [7]. Once married, the pressure to have a baby is often high on young couples because of the economic and social value that is attached to childbearing in most African societies [34]. In the same vein, marriage is a protection from societal stigma for due to the social expectation of childbearing among married women.

Poverty equally plays a crucial role in motivating both teenage marriages and teenage pregnancy. In their qualitative study looking at parallel discourses on teenage pregnancy in rural Zambia, [7, 10] found poverty to be a prominent factor in teenage marriages, both from the parents and young women’s perspective. Parents want to marry off their young girls for cash from bride-wealth while young girls are motivated to leave their parents’ homes because of the failure by parents to meet basic needs such as school fees and other daily necessities. Our study also confirms the role of poverty in explaining teenage pregnancy, as we find that teenagers from wealthier households are less likely to have teenage pregnancies compared to those from poorer households. Similar findings were reported by a previous study on the same population in Zambia [4].

As expected, older teenagers have higher odds of teenage pregnancy than younger ones because they are likely to be more sexually active and exposed and thus being predisposed to teenage pregnancy. The fact that the higher odds of pregnancy among older teenagers remained significant even after controlling for age at sexual debut means that it is an important predictor of teenage pregnancy. It is still possible however, that younger teenagers are less likely to report sexual activities due to social and institutional barriers [35]. A complex interchange of factors is likely to be at play to explain older teenagers and pregnancy. Family and peer pressure to have boyfriends and get married as elucidated by earlier studies, is more likely to be felt the most by older teenagers [23, 36]. Furthermore, older teenagers are more likely to experience parental pressure to be married [1, 37]. Another dynamic, which is likely to make older teenagers to be more exposed to teenage pregnancy, is the fact that they are more likely to live independently from parents, which increases the chance of risky sexual behaviour [21, 22, 38, 39].

Unlike many similar previous studies [3–5] our research does not find a significant relationship between educational level and teenage pregnancy. This study instead, finds literacy to be an important predictor of teenage pregnancy. Illiterate young women have higher odds of getting pregnant compared to literate ones. We suspect that correlation between literacy levels and educational level must have accounted for the non-significance of the latter in this study. It is plausible that literate women would be more empowered with the skills to prevent pregnancy than their illiterate counterparts would. Since literacy is directly associated with educational level, it is reasonable that in Africa, teenagers who are out of school are more likely to get pregnant and have an early start to childbearing than those who are in school [3]. This could also be the reason for high prevalent rates of teenage pregnancy and teenage marriages in sub-Saharan Africa where almost one third of teenagers are out of school [40]. Literate women are expected to have better access to sexual education and be better informed about sexual, health and reproductive rights that help them to avoid early and risky behaviours [41–44].

The results from this study indicate that early sexual debut increases the odds of teenage pregnancy. Sexual debut is a proxy measure of a woman’s onset exposure to pregnancy due to the widespread nature of premarital sex in most societies [45]. Early sexual debut exposes young girls to risks of pregnancy at their most fertile ages, keeps them at risk of pregnancy longer due to their younger ages at sexual debut and elongates their reproductive lifespan compared to their counterparts that initiate sex late especially in relation to inadequate sexual education and contraceptive use [1, 46, 47]. A study in East Africa also had similar findings [26]. In Zambia, some studies have pointed to the role played by social norms and the pressure for material gains as influencing factors for young girls to engage in early sexual debut and subsequently teenage pregnancy [7, 23]. Zambia’s social norms on sexual behaviour are anchored in secrecy and religion. Svanemyr’s (2020) study reported that no parents indicated lack of adequate knowledge on risks of unprotected sex as the reason for teenage pregnancy [23].

Inadequate contraceptive use is also another critical factor that was not mentioned by parents and yet crucial in teenage pregnancy prevention [5]. The secrecy around sex topics between parents and children illuminated by social and cultural norms is a major contributing factor for early sexual debut [3]. Pressure for material benefits has previously been constructed as a parallel and non-dominant narrative of sexual behaviour and teenage pregnancy and yet very common especially in rural Zambia [7, 23].

Media exposure is significantly associated with teenage pregnancy among teenagers in this study. Teenagers who are exposed to FP messages are less likely to have teenage pregnancy. Other studies conducted in SSA found this relationship to be statistically significant [48, 49]. FP messages reinforces the knowledge base of young people on safer sex and contraceptive use which prevents teenage pregnancy. Disseminating of such information has become relatively easier in this era of improved technology where mobile communication gadgets are carried everywhere, coupled with enhanced ways of sharing information through social media and other related platforms.

Surprisingly, knowledge of ovulation period, employment status and recent visits to a health facility were associated with higher odds of teenage pregnancy. These results seem to validate claims of rational choice in teenage pregnancy as made by some scholars [4]. They posit that teenage pregnancy in developing countries may be more intended than in developed countries in view of social and cultural norms that seem to encourage the practice [23, 50, 51]. Our results, which show that young women with knowledge of the ovulation period had higher odds of falling pregnant which pregnancies, could be intentional. Similarly, results, which indicate those employed as being more likely to have teenage pregnancy, may suggest that they rationally decided to get pregnant because of the financial security their employment brings to taking care of children. Several studies have found that working adolescents in SSA are more likely to experience teenage pregnancy compared to those not in employment [52–54].

This study found that variations in teenage pregnancies are mostly attributable to individual level factors. However, some significant variations were also observed between communities. Apart from community FP messages, none of the contextual factors were found to predict teenage pregnancy. Even more startling is the finding that place of residence, which has always been found to be associated with teenage pregnancy was not the case in our study. A Zambian study focusing on a similar population group reported that rural young women are more likely to have teenage pregnancy compared to their urban counterparts [4]. However, our study is significantly different as it pools data from four surveys compared to just one analysed by Munakampe (2021) and colleagues [55]. Findings demonstrate the presence of other factors, which may be more important than residence are influencing sexual activities among young people and subsequently teenage pregnancy.

## Conclusion

This trends analysis shows that teenage pregnancy remains a social and public health challenge in Zambia as the country has experienced little decrease in the years under consideration. The major factors associated with teenage pregnancy include marital status, and employment, knowledge of ovulation period, wealth quintile, early sexual debut and media FP messaging at the individual level. Only community media FP messages was associated with teenage pregnancy among contextual factors. Although, most of the variations in the factors associated with teenage pregnancy in Zambia were attributable to individual level factors, community level characteristics also played a role in explaining the occurrence of teenage pregnancy. Our study provides insights into the importance of considering contextual factors when designing interventions to address teenage pregnancy. The study suggests that early sexual debut and teenage pregnancy are intertwined. Stakeholders need to prioritise the fight against teenage marriage in order to prevent pregnancy in teenagers. Emphasis should be put on improving literacy levels and the general fight against poverty. Considering the fact that social and cultural norms may be preventing parents and children to discuss sex topics in Zambia, it is important to encourage sexual health education promotion through the mass media such as Newspaper, Television (TV), Radio and social media.

### Limitations and strengths of the study

This study had a number of limitations. First, the ZDHS data were collected through a cross-sectional study design that does not permit measurement of causation between outcome of interest and individual and contextual correlates. Second, because our sample was limited to adolescents aged 15–19, our findings cannot be generalized to the broad adolescent age group 10–19 years. Third, our measures of contextual factors represent a proxy that is based on data as captured by the DHS and may not reflect the experience from the communities. Although DHS data cannot facilitate causal inferences, the DHS uses nationally representative sample, which allows for the generalizability of study findings to the entire country based on the study sample, which demonstrates its strength in measuring country-level outcomes important for policy implications. In addition, the rigor of the DHS methodology generally validates the quality of data and outcomes therefrom. The results of this study are comparable across countries where the DHS is conducted. As such, this study makes a significant contribution to the body of knowledge in terms of understanding contextual factors influencing teenage pregnancy in Zambia. Because of lack of longitudinal data in most Africa countries to measure health indicator changes overtime, the DHS data offers a good opportunity to measure repeated measures.

## Supplementary Information

**Additional file 1: Table S1.** Multicollinearity test.

## Data Availability

Data used in our study is publicly available at DHS program website (https://dhsprogram.com/). Data analysis files can be provided upon request to the corresponding author (million.phiri@unza.zm).

## Abbreviations

CI: Confidence interval
DHS: Demographic and Health Survey
EA: Enumeration area
FP: Family planning
SRH: Sexual reproductive health
SSA: Sub-Saharan Africa
UN: United Nations
USAID: United States Aid for International Development
WHO: World Health Organisation
ZDHS: Zambia Demographic and Health Survey
