# Supplementary material for: A multilevel analysis of trends and predictors associated with teenage pregnancy in Zambia (2001–2018)
Source: Reprod Health. 2023 Jan 18;20:16. doi: 10.1186/s12978-023-01567-2 (PMC9848028; doi:10.1186/s12978-023-01567-2)
Supplement: Supplementary file 1 — Additional file 1: Table S1. Multicollinearity test. [file 12978_2023_1567_MOESM1_ESM.docx]

| Table S1: Multicollinearity test |  |
| --- | --- |
| **Variable** | **VIF** |
| Survey year | 1.12 |
| Age | 1.34 |
| Education level | 2.04 |
| Literacy | 1.73 |
| Employment status | 1.03 |
| Wealth status | 2.12 |
| Marital status | 1.23 |
| Knowledge ovulation period | 1.03 |
| Age at first sex | 1.24 |
| Knowledge of FP method | 1.04 |
| Exposure to media FP messages | 1.24 |
| Visited health facility in last 12 months | 1.07 |
| Place of residence | 1.78 |
| Community education | 1.41 |
| Community poverty | 1.66 |
| Community media FP exposure | 1.22 |
| VIF; variance inflation factor |  |
